# Síndrome do PRKAG2: É Efetivo o Rastreio com Ecocardiograma Precoce em Crianças com Genótipo Positivo?

**DOI:** 10.36660/abc.20240543

**Published:** 2024-09-18

**Authors:** Maria Elisa Lucena Sales de Melo Assunção, Norma Lucena Cavalcanti Licínio da Silva, Mychelle Pascoaline de Miranda Silva

**Affiliations:** 1 Universidade de Pernambuco Pronto Socorro Cardiológico de Pernambuco Recife PE Brasil Universidade de Pernambuco – Pronto Socorro Cardiológico de Pernambuco, Recife, PE – Brasil; 2 Instituto Aggeu Magalhães Recife PE Brasil Instituto Aggeu Magalhães – Fiocruz, Recife, PE – Brasil

**Keywords:** Cardiomiopatias, Criança, Genética, Arritmias Cardíacas, Cardiomegalia

Descrita pela primeira vez em 2001 por Gollob et al.,^1^ a síndrome determinada pela mutação do gene PRKAG2 é rara, autossômica dominante, com alta penetrância e prevalência desconhecida na população. Ela se manifesta com o fenótipo de hipertrofia miocárdica e pré-excitação ventricular associada. Taquiarritmias são comuns nessa síndrome genética, assim como distúrbios no sistema de condução cardíaco que levam, em alguns casos, a implante de marca-passo em idade precoce.^2^

A mutação do gene PRKAG2 se apresenta habitualmente como uma fenocópia de cardiomiopatia hipertrófica, diferindo desta por não haver alterações sarcoméricas e, sim, acúmulo de glicogênio nos miócitos cardíacos.^3^ O diagnóstico definitivo é dado pelo estudo genético, mas a apresentação com hipertrofia do ventrículo esquerdo (VE) e distúrbios do sistema de condução fazem diagnóstico diferencial também com as doenças de Danon e de Anderson-Fabry.^4^

Embora a hipertrofia cardíaca seja a manifestação mais prevalente, há a descrição na literatura de cardiomiopatia dilatada e miocárdio não compactado associado à síndrome,^5^ além de acometimento do ventrículo direito.^6^ De início precoce, a cardiomiopatia aparece entre o final da adolescência e início da idade adulta, enquanto os distúrbios de condução, a partir da terceira década de vida. A morte súbita algumas vezes é a manifestação inicial da doença em adultos e crianças afetados.^2,7^

Devido ao seu caráter dominante e alta penetrância, há uma grande probabilidade de filhos de pacientes portadores da doença herdarem o gene e manifestarem a síndrome ao longo da vida, podendo ocorrer, inclusive, no período neonatal.^8^ Buscando fazer o rastreio de crianças que herdaram o gene PRKAG2 dos seus pais, o artigo Achados Ecocardiográficos em Crianças de Pacientes com Diagnóstico de Síndrome do PRKAG2,^9^ comparou o ecocardiograma de 7 crianças assintomáticas com genótipo positivo com o de 7 crianças presumidamente sem a mutação. As crianças avaliadas pelo estudo tinham idades entre 9 meses e 12 anos e foram pareadas por sexo e idade e comparados os parâmetros ecocardiográficos convencionais, ao exame tridimensional e os índices de deformação miocárdica obtidos pela técnica do speckle tracking tanto na modalidade bi como tridimensional.^9^

Estudo inédito na literatura,^9^ encontrou uma diferença estatisticamente significativa quanto à medida do diâmetro anteroposterior do átrio esquerdo (p=0,026), espessura diastólica do septo interventricular (p=0,017), da parede posterior do VE (0,007) e a espessura relativa da parede do VE (p=0,004) cujas medidas foram maiores no grupo de crianças com o genótipo positivo para síndrome do PRKAG2. A massa miocárdica do VE indexada pela superfície corpórea foi maior no grupo de crianças sem presunção de alterações genotípicas (p=0,007).^9^

Sendo o ecocardiograma um dos primeiros exames de imagem a ser solicitado para avaliação de cardiomiopatias, encontrar parâmetros que possam estar alterados de forma precoce em crianças portadoras de alterações no gene PRKAG2 ou sob risco de desenvolver a síndrome devido história familiar de mutação, possibilita um rastreio custo-efetivo numa idade em que não se espera manifestação fenotípica da doença.

Ainda sobre os achados do estudo,^9^ apenas uma criança apresentou alteração ao exame físico com ausculta de sopro sistólico em foco aórtico, mas o artigo não mencionou a etiologia desse sopro, nem se foi uma alteração encontrada no grupo caso ou controle.^9^ Em relação ao eletrocardiograma, todos eram em ritmo sinusal, entretanto, houve dois exames com alterações significativas: PR curto e bloqueio de ramo direito. Como na síndrome do PRKAG2 caracteriza-se por pré-excitação ventricular e distúrbios no sistema de condução, talvez essas alterações eletrocardiográficas encontradas sejam uma manifestação fenotípica precoce, contudo, o artigo não deixou claro se foram alterações encontradas nos pacientes com genótipo positivo para a mutação no PRKAG2.

Diante disso, fica evidente a importância de não apenas termos conhecimento da existência da mutação, mas qual a alteração genética específica encontrada em cada paciente, a idade em que ela se manifesta, qual a apresentação fenotípica inicial e qual a gravidade com que os sinais e sintomas se apresentam visando entender melhor o espectro da doença e planejarmos uma abordagem diagnóstica eficaz com a finalidade de prever e tratar desfechos adversos como morte súbita, taquiarritmias graves ou mesmo bradiarritmias, frequentes nos pacientes com a síndrome do PRKAG2.

Mais estudos são necessários, com um número maior de participantes, para consolidar o ecocardiograma como exame de rastreio tanto em pacientes genótipo positivo e fenótipo negativo para a síndrome do PRKAG2 quanto para aqueles que estão em risco de desenvolver a doença diante de uma história familiar positiva para alteração genética mas que não tiveram acesso ao exame genético. Neste contexto, o estudo^9^ é de fundamental importância como uma evidência inicial para que possamos buscar ativamente alterações cardíacas nas crianças com risco de desenvolver a doença, entender o espectro da doença e, assim, traçar uma estratégia de prevenção de desfechos adversos nessa população.
