# Supplementary material for: Síndrome do PRKAG2: É Efetivo o Rastreio com Ecocardiograma Precoce em Crianças com Genótipo Positivo?
Source: Arq Bras Cardiol. 2024 Sep 18;121(8):e20240543. [Article in Portuguese] doi: 10.36660/abc.20240543 (PMC11495802; doi:10.36660/abc.20240543)
Supplement: Translation

# Translation: PRKAG2 Syndrome: Is Screening with Early Echocardiography Effective in Children with a Positive Genotype?

**DOI:** 10.36660/abc.20240543i

**Authors:** Maria Elisa Lucena Sales de Melo Assunção, Norma Lucena Cavalcanti Licínio da Silva, Mychelle Pascoaline de Miranda Silva

**Affiliations:** 1 Recife PE Brazil Universidade de Pernambuco – Pronto Socorro Cardiológico de Pernambuco, Recife, PE – Brazil; 2 Recife PE Brazil Instituto Aggeu Magalhães – Fiocruz, Recife, PE – Brazil

**Keywords:** Cardiomyopathies, Child, Genetics, Cardiac Arrhythmias, Cardiomegaly

Described for the first time in 2001 by Gollob et al.,^1^ the syndrome determined by the mutation of the PRKAG2 gene is rare, autosomal dominant, with high penetrance and unknown prevalence in the population. It manifests with the phenotype of myocardial hypertrophy and associated ventricular pre-excitation. Tachyarrhythmias are common in this genetic syndrome, as are disturbances in the cardiac conduction system that lead, in some cases, to pacemaker implantation at an early age.^2^

The PRKAG2 gene mutation usually presents as a phenocopy of hypertrophic cardiomyopathy, differing from this in that there are no sarcomeric changes and, rather, accumulation of glycogen in cardiac myocytes.^3^ The definitive diagnosis is given by genetic study, but the presentation with left ventricular (LV) hypertrophy and conduction system disorders also form a differential diagnosis with Danon and Anderson-Fabry diseases.^4^

Although cardiac hypertrophy is the most prevalent manifestation, there is a description in the literature of dilated cardiomyopathy and non-compacted myocardium associated with the syndrome,^5^ in addition to the involvement of the right ventricle.^6^ With an early onset, cardiomyopathy appears between the end of adolescence and the beginning of adulthood, while driving disorders start in the third decade of life. Sudden death is sometimes the initial manifestation of the disease in affected adults and children.^2,7^

Due to its dominant character and high penetrance, there is a high probability that children of patients with the disease will inherit the gene and manifest the syndrome throughout their lives, which may even occur in the neonatal period.^8^ Seeking to screen children who have inherited the PRKAG2 gene from your parents, the artigo Echocardiographic Findings in Children of Patients Diagnosed with PRKAG2 Syndrome,^9^ compared the echocardiogram of 7 asymptomatic children with a positive genotype with that of 7 children presumably without the mutation. The children evaluated in the study were aged between 9 months and 12 years and were matched by sex and age and compared conventional echocardiographic parameters, three-dimensional examination, and myocardial deformation indices obtained by the speckle tracking technique in both two- and three-dimensional modes.^9^

An unpublished study in the literature,^9^ found a statistically significant difference regarding the measurement of the anteroposterior diameter of the left atrium (p=0.026), diastolic thickness of the interventricular septum (p=0.017), the posterior wall of the LV (0.007) and the relative thickness of the LV wall (p= 0.004) whose measurements were higher in the group of children with the positive genotype for PRKAG2 syndrome. LV myocardial mass indexed by body surface was higher in the group of children without presumption of genotypic changes (p=0.007).^9^

As the echocardiogram is one of the first imaging tests to be requested for the evaluation of cardiomyopathies, finding parameters that may be altered early in children with alterations in the PRKAG2 gene or at risk of developing the syndrome due to a family history of mutation, enables screening cost-effective at an age when no phenotypic manifestation of the disease is expected.

Still, on the findings of the study,^9^ only one child presented changes on physical examination with auscultation of a systolic murmur in the aortic focus, but the article did not mention the etiology of this murmur, nor whether it was a change found in the case or control group.^9^ Regarding the electrocardiogram, all were in sinus rhythm; however, there were two tests with significant changes: short PR and right bundle branch block. As PRKAG2 syndrome is characterized by ventricular pre-excitation and disturbances in the conduction system, perhaps these electrocardiographic changes found are an early phenotypic manifestation; however, the article did not make it clear whether these were changes found in patients with a positive genotype for the mutation. in PRKAG2.

In view of this, the importance of not only being aware of the existence of the mutation but also the specific genetic alteration found in each patient, the age at which it manifests itself, what is the initial phenotypic presentation, and the severity of the signs and symptoms is evident. Symptoms are presented in order to understand the spectrum of the disease better and plan an effective diagnostic approach with the purpose of predicting and treating adverse outcomes such as sudden death, severe tachyarrhythmias, or even bradyarrhythmias, common in patients with PRKAG2 syndrome.

More studies are needed, with a larger number of participants, to consolidate echocardiography as a screening test both in genotype-positive and phenotype-negative patients for PRKAG2 syndrome and for those who are at risk of developing the disease due to positive family history. for genetic alteration but who did not have access to genetic testing. In this context, the study of article^9^ it is of fundamental importance as initial evidence so that we can actively look for cardiac changes in children at risk of developing the disease, understand the spectrum of the disease and, thus, outline a strategy to prevent adverse outcomes in this population.
